# The application of DEA (Data Envelopment Analysis) window analysis in the assessment of influence on operational efficiencies after the establishment of branched hospitals

**DOI:** 10.1186/s12913-017-2203-6

**Published:** 2017-04-12

**Authors:** Tongying Jia, Huiyun Yuan

**Affiliations:** Ren Ji Hospital, School of Medicine, Shanghai Jiao Tong University, Shanghai, 200127 China

**Keywords:** DEA window analysis, Multi-campuses hospital, Public hospital, China

## Abstract

**Background:**

Many large-scaled public hospitals have established branched hospitals in China. This study is to provide evidence for strategy making on the management and development of multi-branched hospitals by evaluating and comparing the operational efficiencies of different hospitals before and after their establishment of branched hospitals.

**Methods:**

DEA (Data Envelopment Analysis) window analysis was performed on a 7-year data pool from five public hospitals provided by health authorities and institutional surveys.

**Results:**

The operational efficiencies of sample hospitals measured in this study (including technical efficiency, pure technical efficiency and scale efficiency) had overall trends towards increase during this 7-year period of time, however, a temporary downturn occurred shortly after the establishment of branched hospitals; pure technical efficiency contributed more to the improvement of technical efficiency compared to scale efficiency.

**Conclusions:**

The establishment of branched-hospitals did not lead to a long-term negative effect on hospital operational efficiencies. Our data indicated the importance of improving scale efficiency via the optimization of organizational management, as well as the advantage of a different form of branch-establishment, merging and reorganization. This study brought an insight into the practical application of DEA window analysis on the assessment of hospital operational efficiencies.

## Background

With the rapid economic development in China, the urbanization of rural areas and the scale expansion of modern cities have progressed continuously. Therefore, the demand of medical services, especially high-quality services, becomes bigger and bigger. Under this condition, many large-scaled public hospitals have established branched hospitals in these developing areas to meet the demand of increased population. This type of hospital is increasingly growing in number, for example, more than half of tertiary hospitals have established branched hospital in Shanghai. Is setting up branched hospitals a proper strategy? Will it influence the operational efficiencies of parent hospitals? To answer these questions, we need to find a proper tool for the assessment of operational efficiencies of public hospitals in China, which has not been reported previously. Data Envelopment Analysis (DEA) is a multiple input-multiple output non-parametric evaluation. Due to its enormous advantages in efficiency evaluation in medical and health area, it has already been widely used in the evaluation of hospital efficiency [1]. However, in practical evaluations, researchers will not only pay attention to static operational efficiencies of evaluated unit in a certain period of time, but also focus on dynamic operational efficiencies of the evaluated unit in different periods of time. Meanwhile, it is widely accepted that the number of decision-making unit (DMU) must be large enough to get the significant result [2]. Therefore, the application of DEA was restricted under some circumstances. The DEA window analysis was first put forward by Charnes et al. in 1985 when they tried to analyze the optional efficiency of the United States Air Force Base (USAFB). It is not only applicable to the dynamic operational evaluation with sequential characteristics, but also to the evaluation on small amount of DMUs [3]. These benefits expanded the application of DEA in different areas around the world. In this article, we explored the practical application of DEA window analysis in the evaluation of operational efficiencies before and after the establishment of branched hospitals to provide evidence for strategy making on the management and development of multi-branched hospitals.

## Method

### DEA window analysis

DEA window analysis is based on a dynamic perspective, regarding the same DMU in different period of time as entirely different DMUs. Moving average method is used to choose different reference set in order to determine the relative efficiency of each DMU. That is to say, when the set window slides once, the first period of each window will be deleted and a new period will be added at the same time. The benefit of this method is to describe the dynamic change of the efficiency of each DMU comprehensively, both horizontally and vertically. More importantly, the number of DMU is increased in this method, hence, it enhances the discriminating power by increasing the number of DMUs when a limited number of DMUs is available [4].

Consider a set of N (*n* = 1,…N) DMUs in T (t = 1,…T) period of time. Every DMU has r kinds of input and s kinds of output. Let DMU_n_^t^ denote the level of input or output for DMU n in t period of time, then input vector (X_n_^t^) and output vector (Y_n_^t^) will be presented as [5]:

$$ {X}_n^t=\left[\begin{array}{c}\hfill {x}_n^{\mathrm{l}\; t}\hfill \\ {}\hfill \vdots \hfill \\ {}\hfill {x}_n^{rt}\hfill \end{array}\right]\kern1em {Y}_n^t=\left[\begin{array}{c}\hfill {y}_n^{\mathrm{l}\; t}\hfill \\ {}\hfill \vdots \hfill \\ {}\hfill {y}_n^{st}\hfill \end{array}\right] $$

Consider the window starts at the time point of k (1 ≤ k ≤ T), and the window width is w (1 ≤ w ≤ T-k), then input (X_kw_) and output (Y_kw_) matrix of each window (kw) will be presented as [5]:

$$ {X}_{k w}=\left[\begin{array}{c}\hfill {x}_1^k\hfill \\ {}\hfill {x}_1^{k+1}\hfill \\ {}\hfill \vdots \hfill \\ {}\hfill {x}_1^{k+ w}\hfill \end{array}\kern1em \begin{array}{c}\hfill {x}_2^k\hfill \\ {}\hfill {x}_2^{k+1}\hfill \\ {}\hfill \vdots \hfill \\ {}\hfill {x}_2^{k+ w}\hfill \end{array}\kern1em \begin{array}{c}\hfill \cdots \hfill \\ {}\hfill \cdots \hfill \\ {}\hfill \ddots \hfill \\ {}\hfill \cdots \hfill \end{array}\kern1em \begin{array}{c}\hfill {x}_N^k\hfill \\ {}\hfill {x}_N^{k+1}\hfill \\ {}\hfill \vdots \hfill \\ {}\hfill {x}_N^{k+ w}\hfill \end{array}\right]\kern1em {Y}_{k w}=\left[\begin{array}{c}\hfill {y}_1^k\hfill \\ {}\hfill {y}_1^{k+1}\hfill \\ {}\hfill \vdots \hfill \\ {}\hfill {y}_1^{k+ w}\hfill \end{array}\kern1em \begin{array}{c}\hfill {y}_2^k\hfill \\ {}\hfill {y}_2^{k+1}\hfill \\ {}\hfill \vdots \hfill \\ {}\hfill {y}_2^{k+ w}\hfill \end{array}\kern1em \begin{array}{c}\hfill \cdots \hfill \\ {}\hfill \cdots \hfill \\ {}\hfill \ddots \hfill \\ {}\hfill \cdots \hfill \end{array}\kern1em \begin{array}{c}\hfill {y}_N^k\hfill \\ {}\hfill {y}_N^{k+1}\hfill \\ {}\hfill \vdots \hfill \\ {}\hfill {y}_N^{k+ w}\hfill \end{array}\right] $$

Substituting the above inputs and outputs of DMU_n_^t^ into relevant models will generate the results of DEA window analysis.

Since DEA was first put forward in 1978, it has been utilized in various areas and developed varied models. The two fundamental models are CCR model and BCC model. The former is based on constant return scale (CRS) and the latter is based on variable return scale (VRS). Therefore, CCR model could only give out the technical efficiency (TE) in practical use. However, BCC model further divides technical efficiency into pure technical efficiency (PTE) and scale efficiency (SE) [6]. Technical efficiency (TE) [7] can be divided into two categories: input-guided and output-guided. The former indicates the achievement of given output level by reducing inputs, the latter indicates the achievement of highest output level by using the given inputs. To measure technical efficiency (TE), gap between actual production and production on the boundary of the feasible production set were examined. This set include all technological possibilities of transforming inputs into outputs. A DMU is technically inefficient if production occurs within the interior of this production set. The Pure Technical Efficiency (PTE) [5] measures how a DMU utilizes the resources under exogenous environments; a low PTE implies that the DMU inefficiently manages its resources. The scale efficiency (SE) [5] measures the influence of the hospital scale. If a DMU achieves a low SE, it means that the DMU’s scale size is not proper. Because public hospitals intend to generate the maximum output by using the input from the government, our study chose to use output-guided BCC model to analyze the TE, PTE and SE of multi-branched hospitals.

### Research design

#### Samples and source of data

Samples were collected from five top-level public hospitals in Shanghai, including Shanghai General Hospital, Shanghai Ren-Ji Hospital, Shanghai Hua-Shan Hospital, Shanghai Shu-Guang Hospital and the Obstetrics and Gynecology Hospital affiliated to Shanghai Fu-Dan University. The above model was utilized to evaluate the operational efficiency before and after the establishment of branched hospitals of all these five public hospitals. For pre-period, the operational efficiency of parent hospital was evaluated alone, but parent hospital and branches were examined as a whole for post-period. The source data stem from the business data report provided by Medical and Health Administration Department together with the surveys on all of the institutes. The data of 7 years in total was evaluated in this study, including 3 years before, the current year, and 3 years after the establishment of the branched hospitals.

#### The option of input and output indicators

The option of input and output indicators was based on the previous studies [8–11], and taking the accessibility and accuracy of the data into consideration. Most of researchers have applied indicators including the actual number of beds, staff by the end of the period, the number of patients in out-patient and emergency departments, the number of discharged patients, income, expenditure,bed occupation rate, the average days of hospitalization by the end of the period, ect. To identify a clear distinction between technical efficiency and allocative efficiency, this study did not include the financial indicators (e.g. income and expenditure) into the analysis. Considered the major role of labor and capital in hospitals, we followed the approach of Li [11] with further improvements. In his research, two input indicators (the number of staff; the actual number of beds) and two output indicators (the number of patients in out-patient and emergency departments; the number of discharged patients) were applied. We argue that these indicators are appropriate for hospitals in Chinese mainland. Meanwhile, we selected the average days of hospitalization as the output indicator for the following reasons : The average days of hospitalization has attracted more and more concern in Chinese hospital, many attempts have been made to shorten it, including the great development of ambulatory surgery. The indicator may become one of the much important marks of management level especially in the huge multi-branched hospitals.

Thus, we used the actual number of beds and staff by the end of the period as two indicators of input. Meanwhile, the number of patients in out-patient and emergency departments, the number of discharged patients, as well as the average days of hospitalization by the end of the period were regarded as three output indicators. The detailed description could be seen in Table 1.

**Table 1:** DEA input and output Indicators

Category	Indicators	Defination
Inputs	The actual number of beds	The number of available bed by the end of the period
	The actual number of staff	Registered staff by the end of the period
Outputs	The number of patients in out-patient and emergency departments	The number of patients coming for outpatient and emergency diagnostic services by the end of the period
	The number of discharged patients	The number of discharged patients after hospitalization by the end of the period
	The average days of hospitalization	The number of total bed day/The number of discharged patients

#### The width of the window

The current year of establishment of the branched hospital is set to M, then 3 years before and 3 years after the establishment could be represented as M-3, M-2, M-1, M + 1, M + 2, M + 3. The width of the window may vary between one and all periods in question. In this paper, the width of the window was set to 3 years based on the reported applications, although the reasons are still not clear [12]. Therefore, window 1 contained data of year M-3 to M-1, window 2 was composed of data of year M-2 to M, et cetera. There were totally 5 windows, 15 DMU in each window, giving the total number of DMU 75 [13].

#### Statistical analysis

DEA-Solver PRO 3.0 SPSS13.0 software and Graph Pad Prism 6 were used for data process and analysis.

## Results

### Technical efficiency (TE) of sample hospitals

**Fig. 1:**
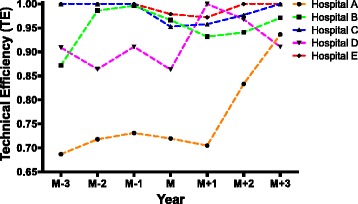
Technical efficiency of sample hospitals before and after the establishment of branched hospitals

Technical efficiency (TE) is the comprehensive evaluation of resource allocation capability and resource utilization efficiency of each DMU. Table 2 and Fig. 1 showed an overall upward tendency of TE in these five hospitals. Except hospital D, technical efficiency was temporarily decreased shortly after the establishment of branched hospitals (year M and M + 1), however, it gradually rise again afterwards (year M + 2 and M + 3). Three years before establishing branched hospitals (M-3, M-2, M-1), only two (C and E) out of five hospitals had reached effective DEA (TE = 1.0000). In year M, DEA was ineffective (TE < 1.0000) and TE was lower than the year before (M-1) for all these 5 hospitals. In year M + 1, only two (C and D) out of five hospitals had TE higher than the current year of establishment of branched hospitals (M), and hospital D reached effective DEA (TE = 1.0000). In year M + 2, only hospital B had a lower technical efficiency compared to year M; while in year M + 3, all these five hospitals had a higher TE than that in year M. The detailed description of TE could be seen in Table 5 in the Appendix.

**Table 2:** Technical efficiency of sample hospitals before and after the establishment of branched hospitals

Hospital	M-3	M-2	M-1	M	M + 1	M + 2	M + 3
A	0.6870	0.7180	0.7313	0.7197	0.7050	0.8333	0.9363
B	0.8720	0.9860	0.9963	0.9663	0.9317	0.9407	0.9710
C	1.0000	1.0000	1.0000	0.9527	0.9577	0.9770	1.0000
D	0.9090	0.8640	0.9107	0.8633	1.0000	0.9680	0.9107
E	1.0000	1.0000	1.0000	0.9787	0.9720	1.0000	1.0000
Mean	0.8936	0.9136	0.9277	0.8961	0.9133	0.9438	0.9636

### Pure technical efficiency (PTE) of sample hospitals

**Fig. 2:**
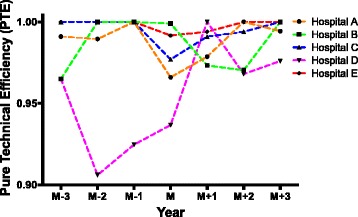
Pure technical efficiency of sample hospitals before and after the establishment of branched hospitals

Pure technical efficiency (PTE) is influenced by factors such as management and techniques. Table 3 and Fig. 2 showed an overall upward tendency of PTE similar to TE in these five hospitals. Once again, except hospital D, PTE was temporarily decreased in year M and gradually increased afterwards. Three years before establishing branched hospitals (M-3, M-2, M-1), only hospital D had not reached effective DEA (PTE = 1.0000). In year M-1, 4 out of 5 hospitals reached effective DEA. However, in year M, DEA was ineffective (PTE < 1.0000) and PTE was lower than the year before (M-1) for all these 5 hospitals. In year M + 1, only hospital B had a lower PTE compared to year M, and hospital D reached effective DEA (PTE = 1.0000). In year M + 2, again only hospital B had a lower pure technical efficiency compared to year M; while in year M + 3, all these five hospitals had a higher PTE than that in year M. The detailed description of PTE could be seen in Table 6 in the Appendix.

**Table 3:** Pure technical efficiency of sample hospitals before and after the establishment of branched hospitals

Hospital	M-3	M-2	M-1	M	M + 1	M + 2	M + 3
A	0.9910	0.9895	1.0000	0.9660	0.9787	1.0000	0.9943
B	0.9650	1.0000	1.0000	0.9990	0.9733	0.9703	1.0000
C	1.0000	1.0000	1.0000	0.9770	0.9910	0.9940	1.0000
D	0.9650	0.9060	0.9247	0.9367	1.0000	0.9680	0.9760
E	1.0000	1.0000	1.0000	0.9917	0.9940	1.0000	1.0000
Mean	0.9842	0.9791	0.9849	0.9741	0.9874	0.9865	0.9941

### Scale efficiency (SE) of sample hospitals

**Fig. 3:**
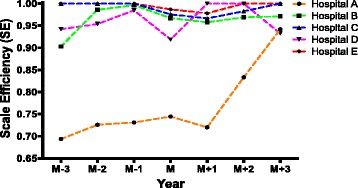
Scale efficiency of sample hospitals before and after the establishment of branched hospitals

Scale efficiency (SE) is influenced by scale of the hospital. In Table 4 and Fig. 3, it demonstrated an overall upward tendency of SE similar to TE and PTE in these five hospitals. Except hospital A, SE was temporarily decreased in year M and gradually increased afterwards. Hospital C and E had effective DEA (SE = 1.0000) in all 3 years before establishing branched hospitals (M-3, M-2, M-1), while other three hospitals had not reached effective DEA in these 3 years. In year M, DEA was ineffective (SE < 1.0000) for all these 5 hospitals, however, only hospital A had an increased SE compared to year M-3, M-2 and M-1. In year M + 1, only hospital D had a higher SE compared to year M and reached effective DEA (SE = 1.0000). In year M + 2 and M + 3, all these five hospitals had a higher SE than that in year M. The detailed description of SE could be seen in Table 7 in the Appendix.

**Table 4:** Scale efficiency of sample hospitals before and after the establishment of branched hospitals

Hospital	M-3	M-2	M-1	M	M + 1	M + 2	M + 3
A	0.6940	0.7260	0.7313	0.7447	0.7203	0.8333	0.9420
B	0.9030	0.9860	0.9963	0.9673	0.9577	0.9690	0.9710
C	1.0000	1.0000	1.0000	0.9757	0.9667	0.9827	1.0000
D	0.9420	0.9540	0.9847	0.9190	1.0000	1.0000	0.9327
E	1.0000	1.0000	1.0000	0.9870	0.9780	1.0000	1.0000
Mean	0.9078	0.9332	0.9425	0.9187	0.9245	0.9570	0.9691

### Comparison between PTE and SE

**Fig. 4:**
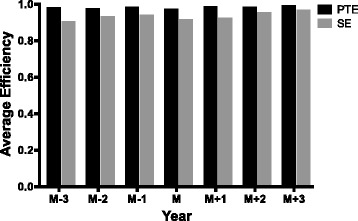
Comparison of the average of pure technical efficiency (PTE) and scale efficiency (SE) of all the sample hospitals before and after the establishment of branched hospitals

When comparing the average of PTE with that of SE, the former value was always higher than the latter value in the whole period of time (7 years) in all of the five sample hospitals, as shown in Fig. 4. Since TE = PTE x SE, therefore, the increase of TE in sample hospitals was mainly dependent on the increase of PTE rather than SE. That is to say, the SE of each sample hospital was to be improved.

## Discussion

### The establishment of branched hospitals did not cause the long-term negative effect on hospital operational efficiency

Our study showed the short-term negative effect of the establishment of branched hospitals on technical, pure technical and scale efficiencies of public hospitals. The operational efficiencies were decreased within one to 2 years after establishing branched hospitals. However, the overall tendency of these efficiencies was towards increase within the whole period of time in this study. Our findings suggested that the scientific planning and assessment of input and output before setting up branched hospitals may help to shorten the period of efficiency optimization. After all, it was beneficial to establish branched hospitals for public hospitals in the long term.

### Multi-branched hospitals should put emphasis on the increase of scale efficiency

The technical efficiency of sample hospitals remained at the high level, in particular, pure technical efficiency contributed more to the high technical efficiency compared to scale efficiency, indicating that public hospitals should put more attention to improve scale efficiency than pure technical efficiency (e.g. management and techniques) when planning the establishment of branched hospitals. In fact, the problem of the scale is actually related to the capability of entrepreneur and the capability of overcoming the decrease of returns-to-scale by the optimization of organizational structure (e.g. distribution of decision-making power, performance assessment, and promotion methods) [14]. Hence, in order to increase the scale efficiency, the multi-branched hospital should take the optimization of organizational structure into consideration.

### Public hospitals could consider various ways including merging and reorganization to establish branched hospitals

Among five sample hospitals, hospital E became a multi-branched hospital through merging and reorganization with other hospitals, whereas other four public hospitals established branched hospitals by building up new buildings. The evaluation of operational efficiencies through our model showed that hospital E had high and stable level of all these efficiencies (TE, PTE and SE) before and after the establishment of branched hospital, while there were larger variations of operational efficiencies happened in other four public hospitals after setting up newly-built branched hospitals. It was indicative that various ways of establishing branched hospitals may lead to the difference in operational efficiencies and the way of merging and reorganization may have some advantages in efficiency optimization.

## Conclusions

This study was the first to report the application of DEA window analysis model in the assessment of operational efficiencies of multi-branched public hospitals in China. Through the comparison of operational efficiencies (including technical efficiency, pure technical efficiency and scale efficiency) before and after the establishment of branched hospitals, our data provided valuable evidence for strategy making on the development and management of multi-branched hospitals when planning to set up new hospitals.

Many researchers have performed DEA approach in hospital efficiency measurement, and found that DEA window analysis showed ideal applicability and great values in hospital efficiency measurement. Harris, et al [2] examined the impacts of horizontal mergers on America hospital’s technical efficiency before and after merger using DEA window analysis. A pretest-posttest study were designed to include the data of 20 hospitals from the pre merger year, merger year, and post merger year. And they found that mergers could increase a hospital’s level of efficiency.

### Limitations

This is a preliminary study to explore the application of DEA window analysis in multi-branched public hospital’s efficiency evaluation in China. And the limitations of this research were listed as below. Firstly, Future study could include more sample hospitals and other non-branched hospitals or private hospitals as a comparison group, as well as using different methodology such as Malmquist productivity index to further test the application of DEA window analysis in characterizing the operational efficiencies of multi-branched hospitals [15]. Secondly, more in-depth analysis was needed to determine the related factors with efficiency,and more specific measures to increase efficiency may should be given. However, our further studies will be carried out to reveal the comprehensive factors which interact with the efficiencies.

## Appendix

**Table 5:** DEA window analysis for technical efficiency over 7 years

		M-3	M-2	M-1	M	M + 1	M + 2	M + 3	Avg
A	Window1	0.6870	0.7000	0.7030					0.6967
	Window2		0.7360	0.7370	0.7230				0.7320
	Window3			0.7540	0.7380	0.7340			0.7420
	Window4				0.6980	0.7070	0.8620		0.7557
	Window5					0.6740	0.8240	1.0000	0.8327
B	Window1	0.8720	0.9720	0.9890					0.9443
	Window2		1.0000	1.0000	0.9540				0.9847
	Window3			1.0000	0.9610	0.9250			0.9620
	Window4				0.9840	0.9660	0.9960		0.9820
	Window5					0.9040	0.9190	1.0000	0.9410
C	Window1	1.0000	1.0000	1.0000					1.0000
	Window2		1.0000	1.0000	0.9330				0.9777
	Window3			1.0000	0.9310	1.0000			0.9770
	Window4				0.9940	0.9500	1.0000		0.9813
	Window5					0.9230	0.9640	1.0000	0.9623
D	Window1	0.9090	0.8640	0.9110					0.8947
	Window2		0.8640	0.9110	0.8020				0.8590
	Window3			0.9100	0.7880	1.0000			0.8993
	Window4				1.0000	1.0000	0.9470		0.9823
	Window5					1.0000	0.9570	0.8610	0.9393
E	Window1	1.0000	1.0000	1.0000					1.0000
	Window2		1.0000	1.0000	0.9660				0.9887
	Window3			1.0000	0.9700	0.9580			0.9760
	Window4				1.0000	0.9580	1.0000		0.9860
	Window5					1.0000	1.0000	1.0000	1.0000
	Avg	0.8936	0.9136	0.9277	0.8961	0.9133	0.9469	0.9722	0.9233

**Table 6:** DEA window analysis for pure technical efficiency over 7 years

		M-3	M-2	M-1	M	M + 1	M + 2	M + 3	Avg
A	Window1	0.9910	0.9910	1.0000					0.9940
	Window2		0.9880	1.0000	1.0000				0.9960
	Window3			1.0000	0.9590	1.0000			0.9863
	Window4				0.9390	0.9790	1.0000		0.9727
	Window5					0.9570	1.0000	1.0000	0.9857
B	Window1	0.9650	1.0000	1.0000					0.9883
	Window2		1.0000	1.0000	1.0000				1.0000
	Window3			1.0000	0.9970	1.0000			0.9990
	Window4				1.0000	0.9810	1.0000		0.9937
	Window5					0.9390	0.9550	1.0000	0.9647
C	Window1	1.0000	1.0000	1.0000					1.0000
	Window2		1.0000	1.0000	1.0000				1.0000
	Window3			1.0000	0.9310	1.0000			0.9770
	Window4				1.0000	0.9730	1.0000		0.9910
	Window5					1.0000	0.9820	1.0000	0.9940
D	Window1	0.9650	0.9060	0.9250					0.9320
	Window2		0.9060	0.9240	0.8890				0.9063
	Window3			0.9250	0.9210	1.0000			0.9487
	Window4				1.0000	1.0000	0.9470		0.9823
	Window5					1.0000	0.9570	0.9460	0.9677
E	Window1	1.0000	1.0000	1.0000					1.0000
	Window2		1.0000	1.0000	0.9750				0.9917
	Window3			1.0000	1.0000	1.0000			1.0000
	Window4				1.0000	0.9820	1.0000		0.9940
	Window5					1.0000	1.0000	1.0000	1.0000
	Avg	0.9842	0.9791	0.9849	0.9741	0.9874	0.9841	0.9892	0.9833

**Table 7:** DEA window analysis for scale efficiency over 7 years

		M-3	M-2	M-1	M	M + 1	M + 2	M + 3	Avg
A	Window1	0.6940	0.7070	0.7030					0.7013
	Window2		0.7450	0.7370	0.7230				0.7350
	Window3			0.7540	0.7690	0.7340			0.7523
	Window4				0.7420	0.7220	0.8620		0.7753
	Window5					0.7050	0.8240	1.0000	0.8430
B	Window1	0.9030	0.9720	0.9890					0.9547
	Window2		1.0000	1.0000	0.9540				0.9847
	Window3			1.0000	0.9640	0.9250			0.9630
	Window4				0.9840	0.9840	0.9960		0.9880
	Window5					0.9640	0.9630	1.0000	0.9757
C	Window1	1.0000	1.0000	1.0000					1.0000
	Window2		1.0000	1.0000	0.9330				0.9777
	Window3			1.0000	1.0000	1.0000			1.0000
	Window4				0.9940	0.9770	1.0000		0.9903
	Window5					0.9230	0.9810	1.0000	0.9680
D	Window1	0.9420	0.9540	0.9850					0.9603
	Window2		0.9540	0.9860	0.9020				0.9473
	Window3			0.9830	0.8550	1.0000			0.9460
	Window4				1.0000	1.0000	1.0000		1.0000
	Window5					1.0000	1.0000	0.9100	0.9700
E	Window1	1.0000	1.0000	1.0000					1.0000
	Window2		1.0000	1.0000	0.9910				0.9970
	Window3			1.0000	0.9700	0.9580			0.9760
	Window4				1.0000	0.9760	1.0000		0.9920
	Window5					1.0000	1.0000	1.0000	1.0000
	Avg	0.9078	0.9332	0.9425	0.9187	0.9245	0.9626	0.9820	0.9388

## Abbreviations

DEA: Data Envelopment Analysis
TE: Technical efficiency
PTE: Pure technical efficiency
SE: Scale efficiency
